# Identification of a Novel NPC1L1 Inhibitor from Danshen and Its Role in Nonalcoholic Fatty Liver Disease

**DOI:** 10.3390/ijms26062793

**Published:** 2025-03-20

**Authors:** Donghai Xia, Xuan Jiang, Xiaomin Xie, Han Zhou, Dongping Yu, Gaowa Jin, Xianlong Ye, Shenglong Zhu, Zhimou Guo, Xinmiao Liang

**Affiliations:** 1 Key Laboratory of Phytochemistry and Natural Medicines, Dalian Institute of Chemical Physics, Chinese Academy of Sciences, Dalian 116023, China; xiadonghai@dicp.ac.cn (D.X.);; 2 University of Chinese Academy of Sciences, Beijing 100049, China; 3 Ganjiang Chinese Medicine Innovation Center, Nanchang 330000, China; 4 Wuxi School of Medicine, Jiangnan University, Wuxi 214122, China

**Keywords:** Danshen, cryptotanshinone, NAFLD, NPC1L1, cholesterol absorption

## Abstract

Danshen, a well-known traditional Chinese medicine (TCM), has gained increasing attention for its protective effects on nonalcoholic fatty liver disease (NAFLD). However, the molecular mechanisms underlying these effects remain to be elucidated. Niemann-Pick C1-like 1 (NPC1L1), a key transporter mediating intestinal cholesterol absorption, has emerged as a critical target for NAFLD treatment. This study aimed to screen for NPC1L1 inhibitors from Danshen and investigate their therapeutic effects on NAFLD. We established a high-throughput screening platform using stable Caco2 cell lines expressing human NPC1L1 (hL1-Caco2) and discovered that tanshinones (Tans), the liposoluble components of Danshen, inhibited NPC1L1-mediated cholesterol absorption in hL1-Caco2 cells. Additionally, Tans treatment reduced hepatic steatosis in high-fat diet (HFD)-fed mice. To identify the active compounds in Tans, activity-oriented separation was performed by integrating the high-throughput screening platform and two-dimensional chromatographic techniques. Ultimately, cryptotanshinone (CTS) was identified as a novel NPC1L1 inhibitor and significantly decreased hepatic steatosis in HFD-fed mice. Molecular docking and dynamics simulation showed that CTS stably bound with NPC1L1, where TRP383 acted as the key amino acid. Taken together, this study demonstrates, for the first time, that CTS, a liposoluble compound from Danshen, is a novel NPC1L1 inhibitor. Our findings suggest that the inhibitory effect of CTS against NPC1L1-mediated intestinal cholesterol absorption may be a potential mechanism, contributing to its alleviation of NAFLD in mice.

## 1. Introduction

Nonalcoholic fatty liver disease (NAFLD), recognized as the hepatic manifestation of metabolic syndrome, is characterized by excessive lipid accumulation in the liver [1,2]. It encompasses two subtypes: nonalcoholic simple fatty liver (NASFL), which may progress to nonalcoholic steatohepatitis (NASH), and NASH itself, which can progress to cirrhosis, hepatocellular carcinoma, and liver-related death [3,4,5]. Nowadays, driven by its complex pathogenesis, NAFLD has become the most common chronic liver disease and is threatening 38% of the global population [6].

Accumulating evidence indicates that cholesterol metabolism plays a key role in the pathogenesis of NAFLD [7,8,9,10,11]. Excess cholesterol is preferentially stored in the liver, where it can impair liver cells through multiple mechanisms such as promoting lipotoxicity, mitochondrial dysfunction, and inflammation, thereby contributing to NAFLD progression [7]. Niemann-Pick C1-like 1 (NPC1L1), a key transporter mediating intestinal cholesterol absorption, has emerged as a critical therapeutic target for metabolic disorders, including NAFLD [12,13,14,15,16,17]. For instance, studies have demonstrated that the genetic deletion or pharmacological inhibition of NPC1L1 reduces hepatic cholesterol content and ameliorates liver steatosis in high-fat diet (HFD)-fed NAFLD mice [13,14]. Additionally, ezetimibe, the only existing NPC1L1 inhibitor, has been shown to improve lipid profiles and reduce liver injury markers in both animal models and patients with NAFLD [15,16]. More recently, natural compounds derived from traditional Chinese medicine (TCM), such as curcumin and diosgenin, have been reported to alleviate NAFLD by modulating NPC1L1 [18,19]. These findings highlight the potential of NPC1L1 inhibition as an effective therapeutic strategy for NAFLD.

TCM shows great potential in modern drug discovery. Danshen (the dried root of *Salvia miltiorrhiza* Burge) is a well-known TCM and has gained increasing attention for its protective effects on NAFLD [20,21,22,23,24,25]. A meta-analysis suggests that Danshen preparations have positive effects on NAFLD, reducing hepatic lipid content and increasing the total effectiveness rate [20]. Several compounds from Danshen have also been reported to affect NAFLD [21,22,23,24,25]. For example, salvianolic acid B, a hydrosoluble compound in Danshen, ameliorates NAFLD by regulating gut microbiota, the LPS/TLR4 signaling pathway, and the NLRP3 inflammasome [22,23]. Tanshinone IIA, a liposoluble compound in Danshen, acts by inhibiting lipogenesis and inflammation and activating TFEB [24,25]. However, due to the multi-component and multi-target nature of TCM, the molecular mechanisms of Danshen remain to be fully elucidated.

Herein, we established a high-throughput screening platform for NPC1L1 inhibitors and identified cryptotanshinone (CTS), a liposoluble compound from Danshen, as a novel NPC1L1 inhibitor through activity-oriented separation. The hepatic steatosis in HFD-fed mice was significantly alleviated by CTS treatment. Our findings suggest that the protective effect of CTS against NAFLD in mice may be achieved by inhibiting NPC1L1-mediated intestinal cholesterol absorption. This study provides a work basis for the future development of NPC1L1 inhibitors in natural products and demonstrates the feasibility of exploring the bioactive molecules in TCM against new therapeutic targets.

## 2. Results

### 2.1. Tans Inhibits NPC1L1-Mediated Intestinal Cholesterol Absorption

**Figure 1:**
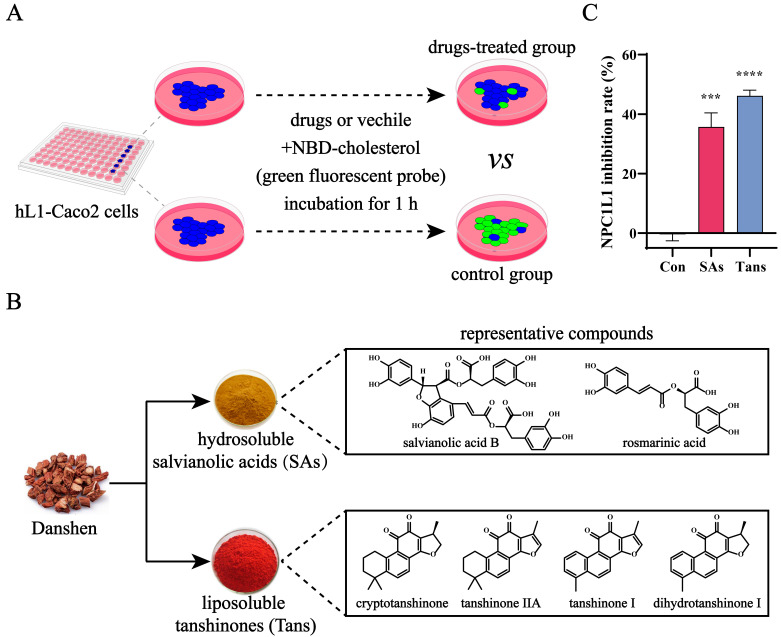
Tans inhibits NPC1L1-mediated cholesterol absorption in hL1-Caco2 cells. (**A**) Working principle of the high-throughput screening platform for NPC1L1 inhibitors. The inhibition activity against NPC1L1 was evaluated by measuring NPC1L1-mediated intestinal cholesterol absorption. Briefly, hL1-Caco2 cells were seeded into 96-well plates and the NBD-cholesterol (green fluorescent probe) absorbed by NPC1L1 was reduced after drug treatment. (**B**) Chemical components of Danshen and structures of the representative compounds. (**C**) Inhibitory effects of the two major groups of active components in Danshen against NPC1L1. SAs, 100 µM; Tans, 8 µM. Data are presented as the means ± SEMs of three independent experiments, each comprising three biological replicates. *** *p* < 0.001, **** *p* < 0.0001 vs. the control group (Con).

NPC1L1 is a key transporter mediating intestinal cholesterol absorption. To screen for NPC1L1 inhibitors in vitro, we successfully established stable Caco2 cell lines expressing human NPC1L1 (hL1-Caco2) (Figure S1). Figure 1A illustrates the working principle of our high-throughput screening platform for NPC1L1 inhibitors. Compared with the control group, the absorption of NBD-cholesterol (green fluorescent probe) mediated by NPC1L1 was blocked, and the fluorescence intensity in hL1-Caco2 cells was reduced when the cells were treated with drugs, indicating an inhibitory effect against NPC1L1. Salvianolic acids (SAs) and tanshinones (Tans), the two major groups of active components in Danshen (Figure 1B), were screened, and Tans exhibited a higher NPC1L1 inhibition rate (Figure 1C). Therefore, Tans was prioritized for further research.

### 2.2. Tans Exhibits a Protective Effect on NAFLD in HFD-Fed Mice

**Figure 2:**
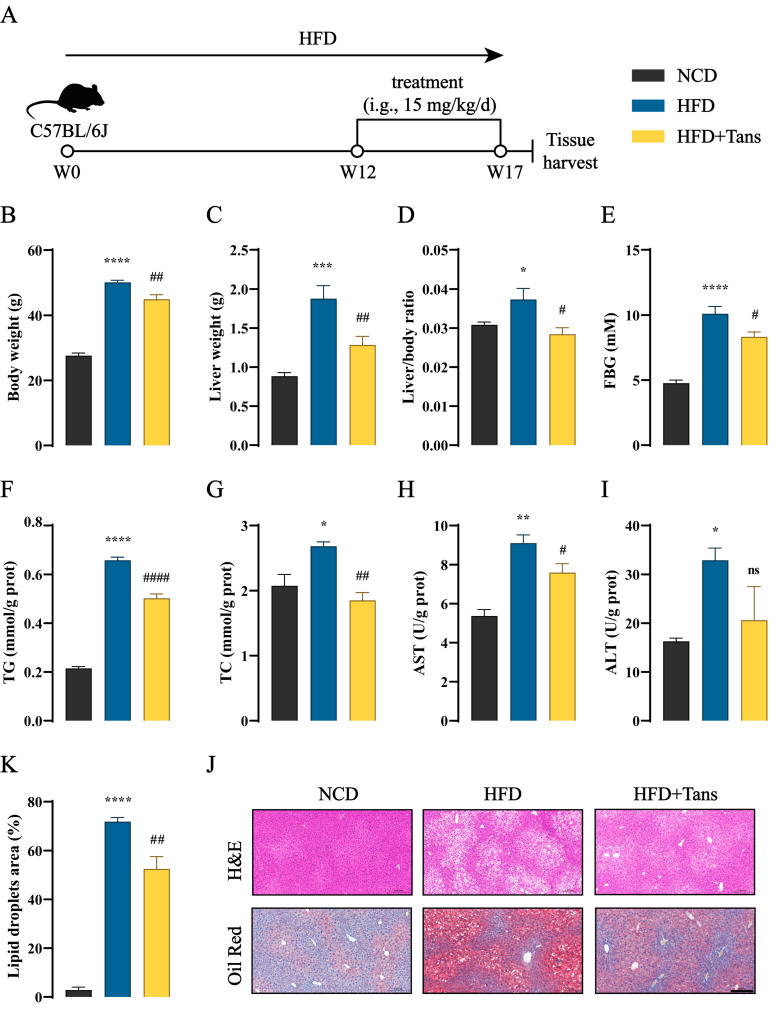
Tans protects against HFD-induced NAFLD in mice. (**A**) Schematic diagram of Tans treatment for NAFLD. C57BL/6J mice fed a high-fat diet (HFD) for 12 weeks, followed by oral administration of Tans (15 mg/kg/d) for another 5 weeks (n = 5). (**B**) Body weight. (**C**) Liver weight. (**D**) Liver/body ratio. (**E**) FBG levels. (**F**–**I**) Hepatic levels of TG (**F**), TC (**G**), AST (**H**), and ALT (**I**). (**J**) Representative H&E and Oil Red O staining of liver sections. Magnification: 200×, Scale bars: 200 µm. (**K**) Lipid droplets area (%). Data are presented as the means ± SEMs (n = 5 per group). * *p* < 0.05, ** *p* < 0.01, *** *p* < 0.001, **** *p* < 0.0001 vs. the normal chow diet (NCD) group. ^#^ *p* < 0.05, ^##^ *p* < 0.01, ^####^ *p* < 0.0001 vs. the HFD group. ns, not significant.

To evaluate the therapeutic effect of Tans on NAFLD in vivo, a high-fat diet (HFD)-fed mouse model was utilized [26]. As shown in Figure 2A, C57BL/6J mice were fed a HFD for 12 weeks, followed by daily oral administration of Tans. In comparison with normal chow diet (NCD), the body weight, liver weight, liver/body ratio, and fasting blood glucose (FBG) were dramatically increased after HFD feeding, while these basic parameters were all significantly reduced after Tans treatment (Figure 2B–E). The hepatic total triglyceride (TG), total cholesterol (TC), aspartate aminotransferase (AST), and alanine transferase (ALT) levels in the HFD group exhibited a significant increase compared to the NCD group (Figure 2F–H). However, the elevated TG, TC, and AST levels were considerably decreased after Tans treatment (Figure 2F–H). Although hepatic ALT levels in the Tans treatment group showed no significant difference with the HFD group, a downward trend was noticed (Figure 2I). Liver hematoxylin–eosin (H&E) and Oil Red O staining revealed that numerous lipid droplets accumulated in the livers of HFD-fed mice, whereas a marked reduction was observed in the Tans treatment group (Figure 2J). Additionally, the lipid droplets area in liver sections stained with Oil Red O was also significantly reduced in the Tans-treated mice compared to those fed HFD (Figure 2K). Taken together, these findings suggest that Tans exhibits a protective effect against HFD-induced hepatic steatosis in mice.

### 2.3. CTS Is the Active Compound in Tans That Inhibits NPC1L1-Mediated Intestinal Cholesterol Absorption

**Figure 3:**
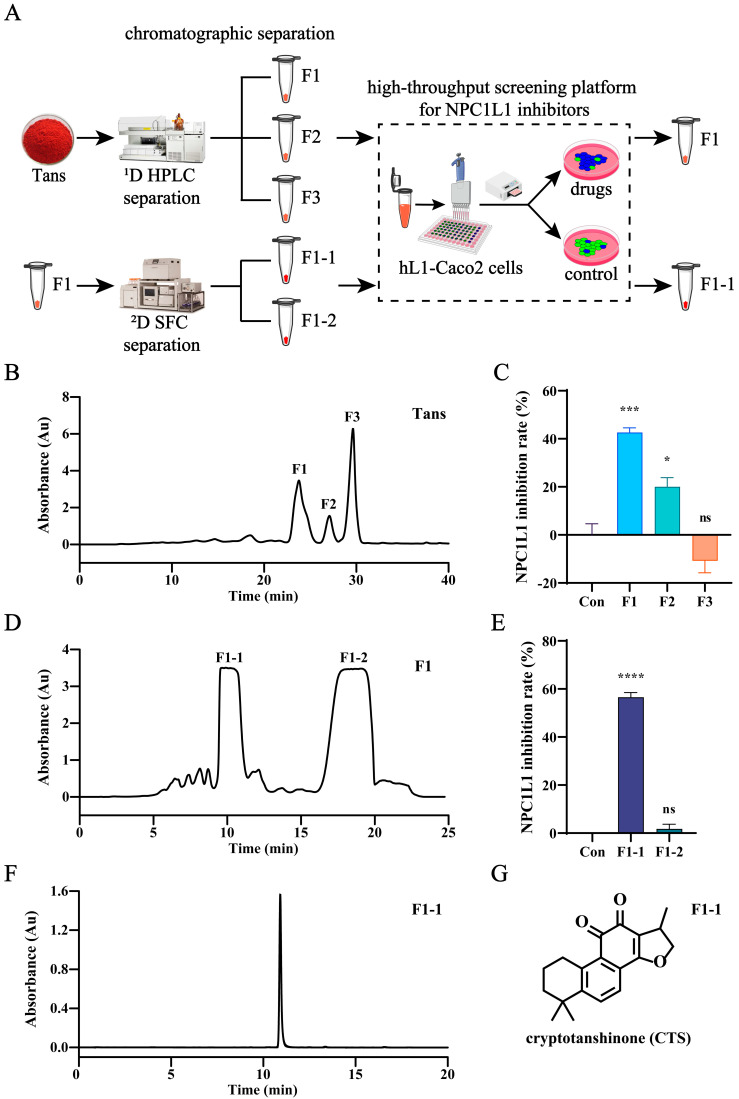
CTS is the active compound in Tans that inhibits NPC1L1-mediated cholesterol absorption in hL1-Caco2 cells. (**A**) The schematic diagram of the screening for NPC1L1 inhibitors in Tans. (**B**) The HPLC separation chromatogram of Tans. (**C**) NPC1L1 inhibition rates of the three major fractions (10 µM) obtained by HPLC separation. (**D**) The SFC separation chromatogram of the fraction F1. (**E**) NPC1L1 inhibition rates of the two major fractions (10 µM) obtained by SFC separation. (**F**) Chromatogram of F1-1. (**G**) Structure of F1-1. Data are presented as the means ± SEMs of three independent experiments, each comprising three biological replicates. * *p* < 0.05, *** *p* < 0.001, **** *p* < 0.0001 vs. the control group (Con). ns, not significant.

To identify the active compounds in Tans, activity-oriented separation was performed by integrating the high-throughput screening platform and two-dimensional chromatographic techniques. Figure 3A illustrates the entire separation and screening process. Briefly, Tans underwent two rounds of chromatographic separation followed by high-throughput screening using hL1-Caco2 cells. Initially, Tans was separated by preparative high performance liquid chromatography (HPLC) and three major fractions (F1, F2, and F3) were collected (Figure 3B). The inhibitory effect of them against NPC1L1 was determined using the high-throughput screening platform. As shown in Figure 3C, both F1 and F2 significantly inhibited NPC1L1 with inhibition rates of 42.6% and 20.1%, respectively, while F3 exhibited a promoting effect on cholesterol absorption. Therefore, F1, the fraction exhibiting the highest inhibition rate, was selected for further separation by semi-preparative supercritical fluid chromatography (SFC) (Figure 3D), and two major fractions (F1-1 and F1-2) were obtained. However, the screening results indicated that only F1-1 exhibited the inhibitory activity (56.6%, Figure 3E). Subsequently, F1-1 was analyzed by HPLC (Figure 3F), liquid chromatography-mass spectrometry (LC-MS) (Figure S2), ^1^H NMR (Figure S3), and ^13^C NMR (Figure S4), and all of these results suggest that F1-1 is the pure substance (HPLC purity 97%), CTS (Figure 3G). Therefore, we conclude that CTS is the active compound in Tans that inhibits NPC1L1-mediated intestinal cholesterol absorption.

### 2.4. CTS Alleviates NAFLD in HFD-Fed Mice

**Figure 4:**
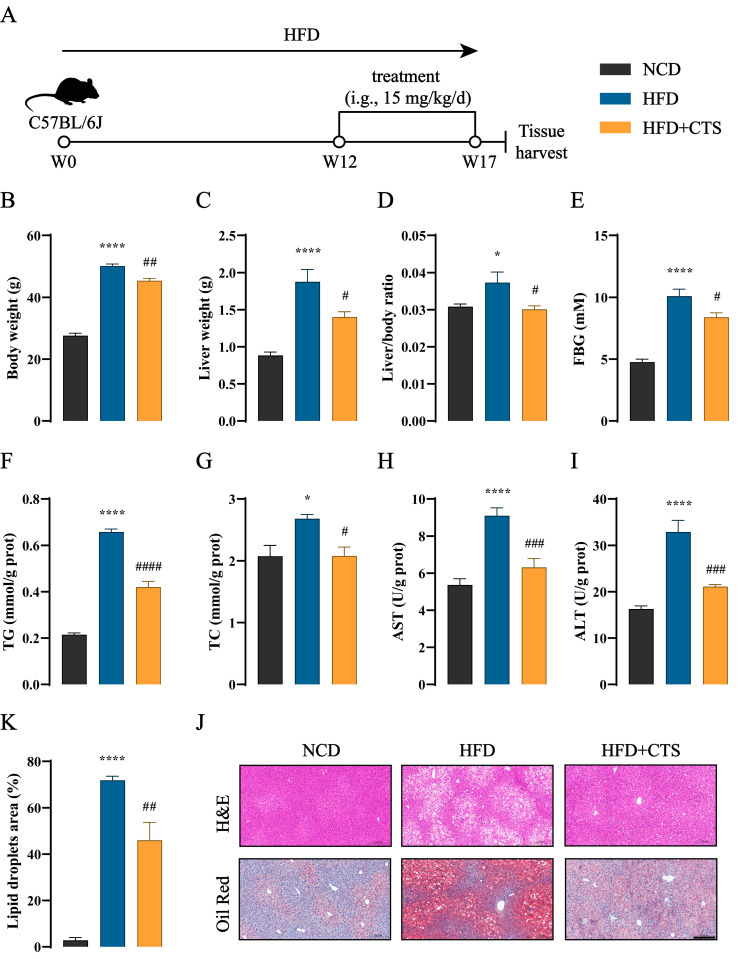
CTS prevents HFD-induced NAFLD in mice. (**A**) Schematic diagram of CTS for NAFLD treatment. C57BL/6J mice were induced by a high-fat diet (HFD) for 12 weeks, followed by oral treatment with CTS (15 mg/kg/d) for another 5 weeks (n = 5). (**B**) Body weight. (**C**) Liver weight. (**D**) Liver/body ratio. (**E**) FBG levels. (**F**–**I**) Hepatic levels of TG (**F**), TC (**G**), AST (**H**), and ALT (**I**). (**J**) Representative H&E and Oil Red O staining of liver sections. Magnification: 200×, scale bars: 200 µm. (**K**) Lipid droplets area (%). Data are presented as the means ± SEMs (n = 5 per group). * *p* < 0.05, **** *p* < 0.0001 vs. the normal chow diet (NCD) group. ^#^ *p* < 0.05, ^##^ *p* < 0.01, ^###^ *p* < 0.001, ^####^ *p* < 0.0001 vs. the HFD group. It should be emphasized that this animal experiment was conducted simultaneously with the Tans treatment. Consequently, the NCD and HFD groups of the two animal experiments in this study shared the same dataset.

The therapeutic effect of CTS on NAFLD in vivo was also evaluated using the HFD-fed mouse model. CTS, the identified active compound, is a major liposoluble component of Danshen and is commercially available. Therefore, the CTS for our animal experiment was commercially obtained. After being induced by HFD for 12 weeks, C57BL/6J mice were gavaged daily with CTS (Figure 4A). As shown in Figure 4B–I, CTS treatment led to a significant decrease in key physiological parameters (body weight, liver weight, liver/body ratio, and FBG) and hepatic biochemical markers (TG, TC, AST, and ALT) in HFD-fed mice. Furthermore, CTS treatment significantly reduced abnormal liver lipid deposition in HFD-fed mice, as demonstrated by the qualitative and quantitative analysis of liver histopathology using liver H&E and Oil Red O staining (Figure 4J,K). Together, these findings reveal that CTS has the potential to alleviate NAFLD in HFD-fed mice.

### 2.5. Molecular Docking and Dynamics Simulation

**Figure 5:**
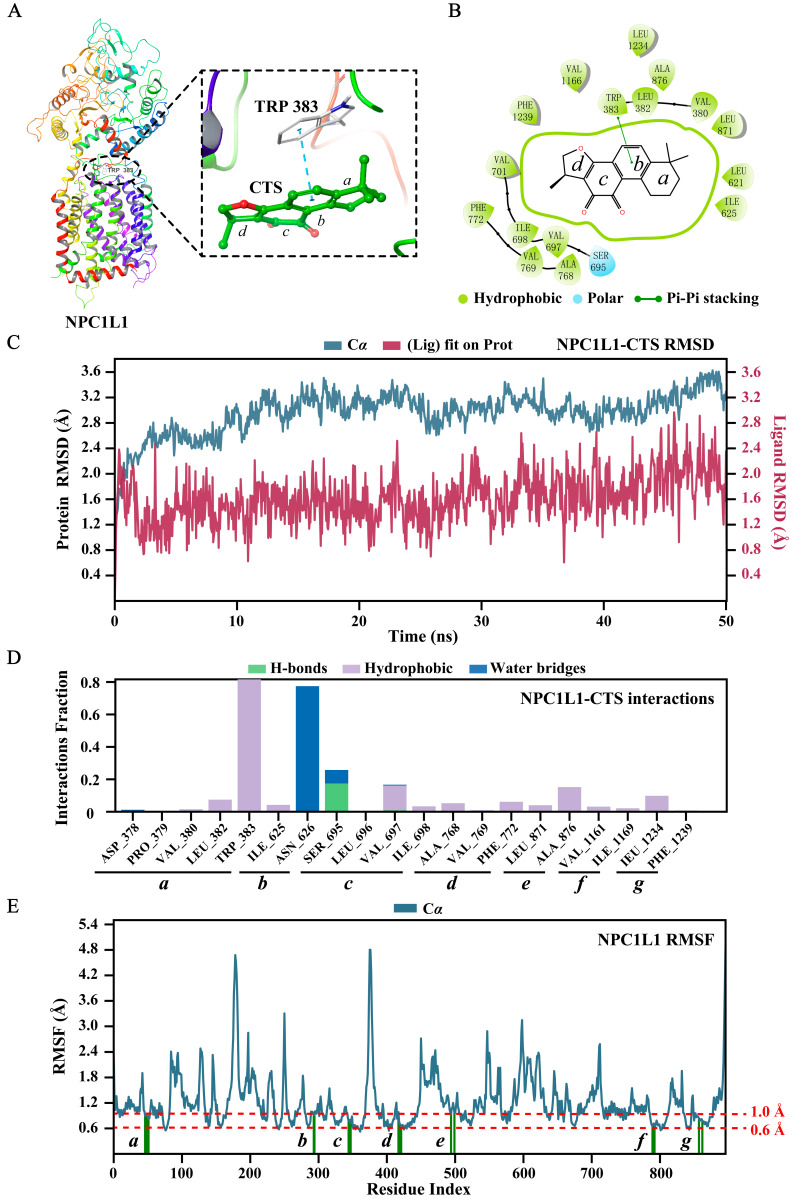
CTS stably binds with NPC1L1. The binding modes of CTS to NPC1L1 (PDB: 7DFZ): (**A**) Three-dimensional presentation. NPC1L1 is represented by a ribbon diagram. CTS is represented by a ball-and-stick model. Carbon atoms are shown in green and oxygen atoms are shown in red. The π-π interaction between CTS and the key amino acid (TRP 383) is displayed using blue dashed lines; (**B**) Two-dimensional presentation. The thin green line marks the π-π interaction between CTS and TRP 383. The thick green circle shows the hydrophobic interaction between CTS and amino acids in the docking pocket. The molecular dynamics (MD) simulation of the NPC1L1-CTS complex: (**C**) Root mean square deviation (RMSD) plots of NPC1L1 (blue, left Y-axis) and CTS (red, right Y-axis); (**D**) NPC1L1-CTS interactions; (**E**) Root mean square fluctuation (RMSF) plot of NPC1L1. NPC1L1 protein residues that interact with CTS are marked with green vertical bars.

To investigate the binding modes of CTS to NPC1L1 (PDB: 7DFZ), we conducted molecular docking using Schrödinger 2021-2. As displayed in Figure 5A, the benzene ring of TRP383 played a key role in the binding, forming a π-π interaction (blue dashed lines) with the benzene ring of CTS. Moreover, hydrophobic interactions (thick green circle, Figure 5B) were observed between CTS and amino acids within the binding pocket, attributed to the hydrophobic structure of CTS. The docking score was –9.267, and the binding energy was –80.92 kcal/mol.

To assess the binding stability of the NPC1L1-CTS complex, we performed a 50-ns molecular dynamics (MD) simulation using the Desmond module of the Schrödinger suite. Root mean square deviation (RMSD) is a crucial parameter in MD simulation analysis, which provides valuable insights into conformational changes and the equilibrium state of the simulated system. As shown in Figure 5C, the RMSD plots of NPC1L1 (blue, left Y-axis) and CTS (red, right Y-axis) increased rapidly and fluctuated only within a small range (both less than 3 Å), indicating that the NPC1L1-CTS complex rapidly reached equilibrium and the conformations of them remained stable. More importantly, the RMSD plot of CTS was lower than that of NPC1L1, showing that CTS was always located in the binding pocket without escaping throughout the simulation. NPC1L1-CTS interactions in the MD simulation were also monitored and presented in Figure 5D. It was obviously observed that the π-π interaction (classified as a hydrophobic interaction in MD simulation) between CTS and TRP383 was maintained nearly 80% of the simulation time, demonstrating the key role of TRP383 in the binding. Root mean square fluctuation (RMSF) is useful for characterizing local changes along the protein chain. NPC1L1 protein residues interacting with CTS were divided into seven regions (*a*–*g*, Figure 5D) and marked with green vertical bars in the RMSF plot of NPC1L1 (Figure 5E). The minor fluctuations (0.6 Å to 1 Å, Figure 5E) indicated that these amino acid residues were stable during the simulation. In summary, our molecular docking and dynamics simulation results demonstrate that CTS stably binds with NPC1L1 and TRP383 is the key amino acid.

## 3. Discussion

**Figure 6:**
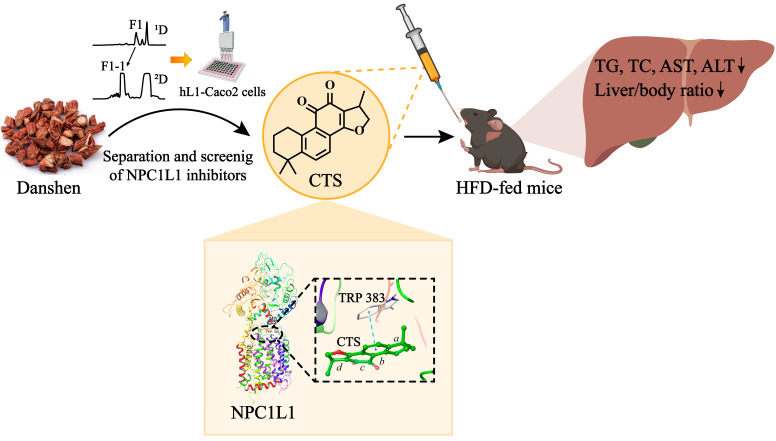
Schematic diagram of the study. CTS, the liposoluble compound in Danshen, was identified as a novel NPC1L1 inhibitor and has a positive effect on NAFLD in HFD-fed mice. Accordingly, CTS may alleviate NAFLD in mice by inhibiting NPC1L1-mediated intestinal cholesterol absorption.

In this study, we developed a high-throughput screening method for NPC1L1 inhibitors and explored the active compounds in Danshen by activity-oriented separation. Ultimately, CTS, the liposoluble compound in Danshen, was identified as a novel NPC1L1 inhibitor and significantly alleviated hepatic steatosis in HFD-fed NAFLD mice, suggesting that CTS may exert its protective effect against NAFLD by inhibiting NPC1L1 (Figure 6).

The development of NPC1L1 inhibitors encounters many challenges, and only one inhibitor (ezetimibe) has been approved so far [27]. NPC1L1 is a 13-pass trans-membrane receptor protein with 1332 amino acids, making it extremely difficult to obtain the intact protein, which limits the screening of its inhibitors at the protein level. Although the crystal structure of NPC1L1 was revealed in 2020 [28], its expression and purification remain challenging. However, the elucidated crystal structure will provide great help for virtual screening and binding studies of candidates with NPC1L1. The cell lines expressing NPC1L1 can be used for the screening of NPC1L1 inhibitors, such as Caco2 and HepG2 cells [18,29]. It is essential to study the effects of candidates on the function of NPC1L1-mediated cholesterol absorption. [^3^H]-cholesterol is the optimal tracer; it is radioactive and the assay cannot be performed in a conventional laboratory [30]. NBD-cholesterol labeled with green fluorescence is becoming an effective substitute for [^3^H]-cholesterol to trace cholesterol absorption in cells [18,29]. Thus, for the high-throughput screening of NPC1L1 inhibitors, we constructed the stable Caco2 cell lines expressing hNPC1L1 (hL1-Caco2) and used NBD-cholesterol as the probe. The binding modes and stability between candidates and NPC1L1 were confirmed through molecular docking and dynamics simulation based on the elucidated crystal structure of NPC1L1.

For the development of active compounds in traditional Chinese medicine (TCM), effective separation of TCM and high-throughput screening are equally important. TCM has to suffer multiple rounds of screening and separation owing to its highly complex chemical components. Chromatographic techniques have demonstrated powerful separation capabilities in TCM [31]. The optimization of chromatographic modes, stationary phases, and mobile phases can provide comprehensive solutions for the multi-dimensional separation of TCM. Herein, to identify the active compounds in Danshen, activity-oriented separation was performed by two-dimensional chromatographic techniques (RPLC and SFC), and CTS was finally identified as a novel NPC1L1 inhibitor.

NPC1L1 mediates intestinal cholesterol absorption, and cholesterol flows in the small intestine and the liver by enterohepatic circulation [18]. It is well established that excess cholesterol accelerates the progression of NAFLD [7]. Accordingly, in principle, it makes sense to alleviate NAFLD by inhibiting NPC1L1, which has been demonstrated in previous studies [12,13,14,15,16,17]. The possible mechanisms for inhibiting NPC1L1 to alleviate NAFLD have also been proposed. It was believed that NPC1L1 inhibition reduced hepatic cholesterol content and cholesterol-dependent activation of liver X receptor, a nuclear receptor promoting hepatic lipogenesis [13]. In our study, CTS, the novel NPC1L1 inhibitor, significantly alleviated hepatic steatosis in HFD-fed mice, indicating that the therapeutic effect of CTS against NAFLD may be regulated by inhibiting NPC1L1-mediated intestinal cholesterol absorption.

There may be some possible limitations in this study. CTS demonstrates a range of beneficial effects, including anti-tumor, cardioprotective, neuroprotective, visceral protective, and anti-inflammatory actions, primarily through the inhibition of NF-κB and MAPK signaling pathways [32]. In addition to these effects, CTS also suppresses the synthesis of pro-inflammatory cytokines such as TNF-α, IL-1, IL-6, IL-8, and IL-17, and it plays a regulatory role in autophagy [33]. All of these mechanisms are involved in the pathogenesis of NAFLD. Therefore, NPC1L1 inhibition may represent only one of the mechanisms through which CTS exerts its therapeutic effect on NAFLD. The specificity of the NPC1L1 pathway, the extent to which its inhibition contributes to NAFLD protection, and the precise mechanisms through which CTS modulates liver lipid metabolism through NPC1L1 inhibition all require further clarification. Additionally, it is worth noting that the tissue expression of NPC1L1 varies across species. In humans, NPC1L1 is predominantly expressed in the small intestine and liver, whereas in mice, its expression is largely confined to the small intestine [34,35]. Thus, the current study cannot be fully translated to humans, and different NAFLD models are needed to evaluate the clinical feasibility of CTS. The HFD-fed hamsters may be an ideal choice, considering their similarities to humans in lipid metabolism and the tissue distribution pattern of NPC1L1 expression [18,36].

Ezetimibe is recognized to inhibit NPC1L1 function by blocking its internalization [37,38]. Our preliminary study revealed that, similar to ezetimibe, CTS did not affect the mRNA level of NPC1L1 but modulated its biological function. The inhibitory effect of CTS against NPC1L1 in hL1-Caco2 cells is slightly lower than that of ezetimibe (IC50 value, 12.35 µM vs. 0.35 µM). Thus, we intend to optimize the structure of CTS to enhance its water solubility and bioavailability and then investigate its pharmacokinetics in animal models. We will also evaluate its long-term efficacy and safety in NAFLD models and conduct an in-depth comparison with ezetimibe to fully validate the therapeutic potential of CTS. Moreover, given its structural similarity with other tanshinones, the potential off-target effects of CTS will be studied using in silico, in vitro, and cell-based experiments.

Despite the limitations discussed above, these concerns are essential considerations in the broader context of drug development. They highlight the need to fully elucidate the action mechanism of CTS and demonstrate its potential for clinical application. However, these considerations do not detract from the novelty and completeness of the present study. On the contrary, they provide valuable insights that will guide our future research directions.

## 4. Materials and Methods

### 4.1. Reagents and Materials

Tanshinones (Tans), the liposoluble components of Danshen, were provided by Xi’an Hao-Xuan Bio-Tech Co., Ltd. (Xi’an, China). Cryptotanshinone (CTS, cat. BP0412, HPLC purity 97%), the liposoluble compound purified from Danshen, was purchased from Chengdu Biopurify Phytochemicals Ltd. (Chengdu, China). The probe molecule 22-(N-(7-nitrobenz-2-oxa-1, 3-diazol-4-yl)-labeled cholesterol (NBD-cholesterol) and reagents for liquid chromatography-mass spectrometry (LC-MS) were obtained from Thermo Fisher Scientific (Waltham, MA, USA). Other relevant reagents for chromatographic separation and analysis were all purchased from Meryer Chemical Technology Co., Ltd. (Shanghai, China).

### 4.2. Animal Experiments

Male C57BL/6J mice (6–8 weeks old, 20 ± 2 g) were purchased from GemPharmatech Co. Ltd. (Nanjing, China) and housed in a specific pathogen-free condition at 25 °C with a 12 h light/dark cycle. After 1 week of acclimatization, C57BL/6J mice were fed either a high fat diet (60% fat, cat. TP23300, Trophic Animal Feed High-tech Co., Ltd., Nantong, China) or a normal chow diet (10% fat, cat. SWS9102, Jiangsu Xietong Pharmaceutical Bio-engineering Co., Ltd., Nanjing, China) for 12 weeks and then mice were randomly assigned to four groups (n = 5): normal chow diet group (NCD), high fat diet group (HFD), high fat diet with Tans treatment group (HFD + Tans), or high fat diet with CTS treatment group (HFD + CTS). The drugs were dissolved in 0.5% carboxy-methyl-cellulose sodium (CMC-Na, Solarbio, Beijing, China) and administered daily via intragastric gavage with 15 mg/kg for 5 weeks. The dose employed in this study was based on a previous study regarding the effectiveness of CTS in C57BL/6J mice with atherosclerosis [39]. The NCD and HFD groups were orally administered 0.5% CMC-Na alone. Food and water were available ad libitum. After 12 h of fasting, all mice were anesthetized and sacrificed. The liver tissues were collected for further analysis.

The animal experiment was approved by the Experimental Animal Ethics Committee of Jiangnan University (JN.No20231007c1400430[468]). All animal experimental procedures followed the Guide for the Care and Use of Laboratory Animals.

### 4.3. Establishment of Stable Caco2 Cell Lines Expressing Human-NPC1L1 (hL1-Caco2) and Cell Culture

The plasmid expressing human-NPC1L1 (hNPC1L1, NCBI Reference Sequence: NM_001101648.2) was constructed by Shenzhen BORUI Pharmaceutical Technology Co., Ltd. (Shenzhen, China). The CDS of hNPC1L1 was connected to the lentiviral vector EDV0005 containing the puromycin resistance (puroR) gene. The Kozak sequence was added to the 5′ end and the 3 × Flag tag was added to the 3′ end. The recombinant plasmid was packaged with lentivirus and then transfected to Caco2 cells. The polyclonal cell pools were screened with 2 µg/mL puromycin (Meilunbio, Dalian, China) for two weeks. After limiting dilution, single-cell clones were generated and expanded to obtain monoclonal cell lines. The resulting stable Caco2 cell lines expressing human-NPC1L1 (hL1-Caco2) were cultured in high glucose Dulbecco’s modified Eagle’s medium (Thermo Fisher Scientific, Waltham, MA, USA) supplemented with 10% fetal bovine serum (VivaCell Biosciences, Shanghai, China) and 1% penicillin-streptomycin (Meilunbio, Dalian, China) at 37 °C with 5% CO_2_.

### 4.4. Biochemical Analysis

Levels of hepatic total triglyceride (TG), total cholesterol (TC), aspartate aminotransferase (AST), and alanine transferase (ALT) were measured using commercial kits (Nanjing Jiancheng, China). Briefly, liver tissues (80–100 mg) were homogenized in cold PBS at a ratio of 1:9 (*w*/*v*). The homogenate was centrifuged (2500 rpm, 4 °C, 10 min) and the resulting supernatant was diluted to determine the above indexes according to the manufacturer’s instructions.

### 4.5. Pathological Analysis

The liver was collected and fixed in 4% paraformaldehyde. The paraffin-embedded sections were subjected to hematoxylin–eosin (H&E) staining. The optimal cutting temperature-embedded sections were subjected to Oil Red O staining. The stained images were scanned by Panoramic MIDI (3D HISTECH, Budapest, Hungary) and presented at 200-fold magnification. The percentage of the Oil Red O-positive staining image area to the total image area was assessed by Aipathwell software (Servicebio, Nanchang, China).

### 4.6. High-Throughput Screening of NPC1L1 Inhibitors

The high-throughput screening of NPC1L1 inhibitors was conducted by Shenzhen BORUI Pharmaceutical Technology Co., Ltd. (Shenzhen, China). The inhibitory activity against NPC1L1 was evaluated by NPC1L1-mediated intestinal cholesterol absorption in hL1-Caco2 cells. NBD-cholesterol with green fluorescence was used as the probe molecule to indicate free cholesterol absorbed by NPC1L1. In brief, hL1-Caco2 cells were seeded into a 96-well plate at a density of 4 × 10^4^ cells/well and cultured until 100% confluence. The cells were then incubated with NBD-cholesterol (20 μg/mL) and indicated drugs for 60 min. The cellular mean fluorescence intensity (content of NBD-cholesterol absorbed by NPC1L1) at 0 min (*F*_0_) and 60 min (*F*_60_) was recorded by a ELx-800 microplate reader (BioTek, Santa Clara, CA, USA) at λ_Ex/Em_ 485/535 nm. The slope of fluorescence change (absorption rate of NBD-cholesterol by NPC1L1) was normalized by the control group and defined as the NPC1L1 inhibition rate.

$$NPC1L1\;inhibition\;rate\;(\%)=-\frac{slope\;of\;treatment\;-\;slope\;of\;control}{slope\;of\;control}\times 100$$

$$slope=\frac{F_{60}-F_{0}}{60}$$

### 4.7. Separation and Analysis of Tans and Related Fractions

The activity-oriented separation of Tans was performed by off-line two-dimensional chromatography. A C18HD column (50 × 250 mm, 10 µm, 100 Å, Acchrom, Beijing, China) was used for the first dimensional (^1^D) separation on a preparative high performance liquid chromatography (HPLC) system (Auto-P, Waters, Milford, MA, USA). Tans was dissolved in MeOH at a concentration of 10 mg/mL and 10 µL of the solution was injected to the system. The elution was carried out with 70~100% MeOH/H_2_O (*v*/*v*) for 40 min at a flow rate of 80 mL/min, and the three most abundant fractions at 270 nm were collected.

The second dimensional (^2^D) separation was accomplished on a semi-preparative supercritical fluid chromatography (SFC) system (Investigator, Waters, Milford, MA, USA) using a ChiralGel BH column (10 × 250 mm, 7 µm, 100 Å, Acchrom, Beijing, China) at 35 °C. An automated back-pressure regulator was set as 100 bar. Dichloromethane was used to dissolve the active fraction F1 (50 mg/mL), 200 µL of which was injected. The mobile phase was composed of CO_2_ and MeOH, and a gradient program was employed as follows: 0~12 min, 5~20% MeOH; 12~18 min, 20% MeOH; 18.1~21 min, 65% MeOH; and 21.1~25, 5% MeOH. The flow rate was 12 mL/min. The two most abundant fractions at 270 nm were collected.

LC-MS analysis of the active fraction F1-1 was conducted on an UPLC-QE Plus system (Thermo Fisher Scientific, Waltham, MA, USA). A BEH Phenyl column (2.1 × 150 mm, 1.7 µm, Waters, Milford, MA, USA) was used for the chromatographic separation at 35 °C. The mobile phase consisted of (A) 0.1% FA/H_2_O (*v*/*v*) and B (ACN), and the elution was carried out with 35~50% B for 20 min at a flow rate of 0.4 mL/min. The sample (F1-1) was dissolved using ACN at a concentration of 10 ppm and the injection volume was 0.3 µL. Full MS/dd-MS^2^ signal in positive ion mode was monitored with a scan range of 200~1500 *m*/*z*. Other MS parameters were set as default values.

The ^1^H and ^13^C NMR spectra of F1-1 were investigated using chloroform-*d* and recorded on a Bruker Avance III-400 spectrometer (Bruker BioSpin AG, Fällanden, Switzerland).

### 4.8. Molecular Docking and Molecular Dynamics (MD) Studies

The co-crystal structure of NPC1L1 was obtained from the Protein Data Bank (PDB: 7DFZ) and then prepared using Schrödinger 2021-2 (Schrödinger, LLC, New York, NY, USA). The protein Prepare Wizard module was employed to hydrogenate the complex, remove crystal waters, and repair loop regions. The energy minimization was carried out by optimized potentials for a liquid simulations-2005 force field. The docking protein grid (10 Å × 10 Å × 10 Å) centered on the original ligand was generated using the Receptor Grid Generation module. Subsequently, the original ligand was removed and CTS was docked into the prepared structure using the Glide module. The binding free energy was calculated using the molecular mechanics-generalized Born surface area method.

A 50-ns molecular dynamics (MD) simulation was performed to confirm the binding stability of the NPC1L1-CTS complex using the Desmond module of the Schrödinger suite. The protocol was referenced to our previous study [40].

### 4.9. Statistical Analysis

Data were analyzed using Origin Pro 2021 (OriginLab Corporation, Northampton, MA, USA) and GraphPad Prism 9 (GraphPad Software Inc., San Diego, CA, USA). The results were presented as the mean ± SEM. A student’s *t* test and one-way ANOVA were employed for two groups and multiple groups comparison, respectively. *p <* 0.05 was considered statistically significant.

## 5. Conclusions

This study successfully established an efficient in vitro high-throughput screening platform for NPC1L1 inhibitors. Using this platform, combined with integrated chromatographic separation techniques, we identified CTS as a novel NPC1L1 inhibitor from Danshen. The in vivo experiment demonstrated that CTS treatment significantly alleviated hepatic steatosis in HFD-fed mice. Consequently, our findings suggest that CTS may alleviate NAFLD in mice by inhibiting NPC1L1-mediated intestinal cholesterol absorption. Furthermore, this screening platform provides a valuable technical reference for future discovery and the development of NPC1L1 inhibitors in natural products.

## Data Availability

The data supporting this study’s findings are available upon request.

## Supplementary Materials

The supporting information can be downloaded at: https://www.mdpi.com/article/10.3390/ijms26062793/s1.
