# Supplementary material for: Identification of a Novel NPC1L1 Inhibitor from Danshen and Its Role in Nonalcoholic Fatty Liver Disease
Source: Int J Mol Sci. 2025 Mar 20;26(6):2793. doi: 10.3390/ijms26062793 (PMC11942890; doi:10.3390/ijms26062793)
Supplement: Supplementary file 1 [file ijms-26-02793-s001.zip › ijms-3470988-supplementary.pdf]

Supplementary Materials and Methods

quantitative real-time PCR (qRT-PCR) Assay

Total RNA was extracted using FastPure cell/tissue total RNA isolation kit V2 (cat. RC112-01, Vazyme, Nanjing, China) and reverse transcribed into cDNA utilizing HiScript III 1st strand cDNA synthesis kit (cat. R312-01, Vazyme, Nanjing, China). mRNA expressions were determined by qRT-PCR (LightCycler 480 II, Roche, Basel, Switzerland) using Hieff qPCR SYBR Green Master Mix (cat. 11201ES08, Yeasen, Shanghai, China). Relative mRNA levels were calculated by $2^{-\Delta\Delta CT}$ method. The primers for qPCR were as follows: human NPC1L1 forward primer (hNPC1L1-F), 5'-GCTGGTGCCATCAGCAACTTC-3', and reverse primer (hNPC1L1-R), 5'-CAGCTC GGTGACCCGCTC-3'. Human GAPDH was used as an internal control with specific primers 5'-TCGACAGTCAGCCGCATCTTCTTT-3' (hGAPDH-F), and 5'-ACCAAA TCCGTTGACTCCGACCTT-3' (hGAPDH-R).

Supplementary Figures

A

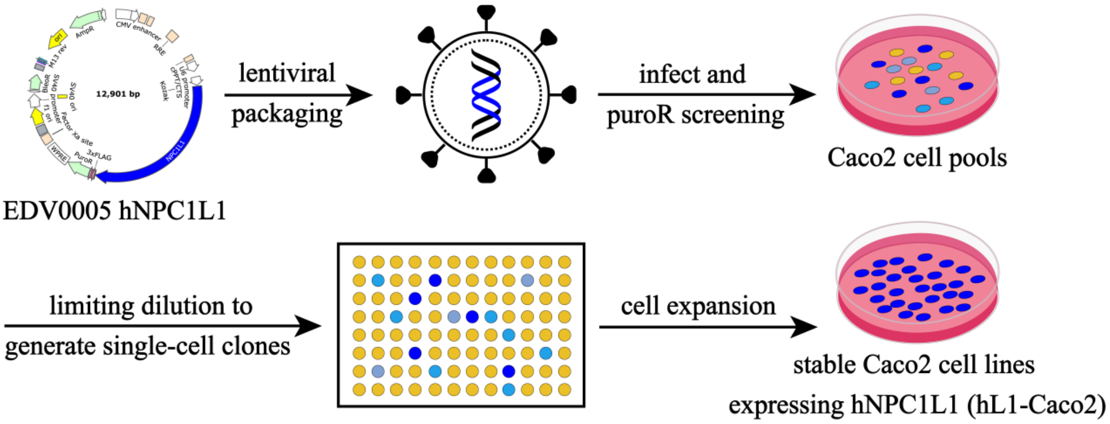

B

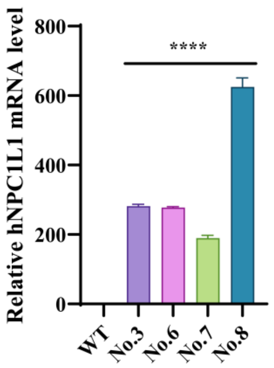

Supplementary Figure S1. Establishment of stable Caco2 cell lines expressing hNPC1L1 (hL1-Caco2). (A) The schematic diagram of the experiment. Briefly, Caco2 cells were infected by lentivirus carrying hNPC1L1 gene and then screened with 2 μg/mL puromycin. The resulting polyclonal cell pools were subjected to limiting dilution and expanding to obtain the stable monoclonal cell lines, designated as hL1-Caco2. (B) mRNA levels of hNPC1L1 in four different monoclonal cell lines. Cell line No.8, exhibiting the highest hNPC1L1 expression, was selected for further use. Data are presented as the means±SEMs (n=2 per group). $^{****}p < 0.0001$ vs. the WT group.

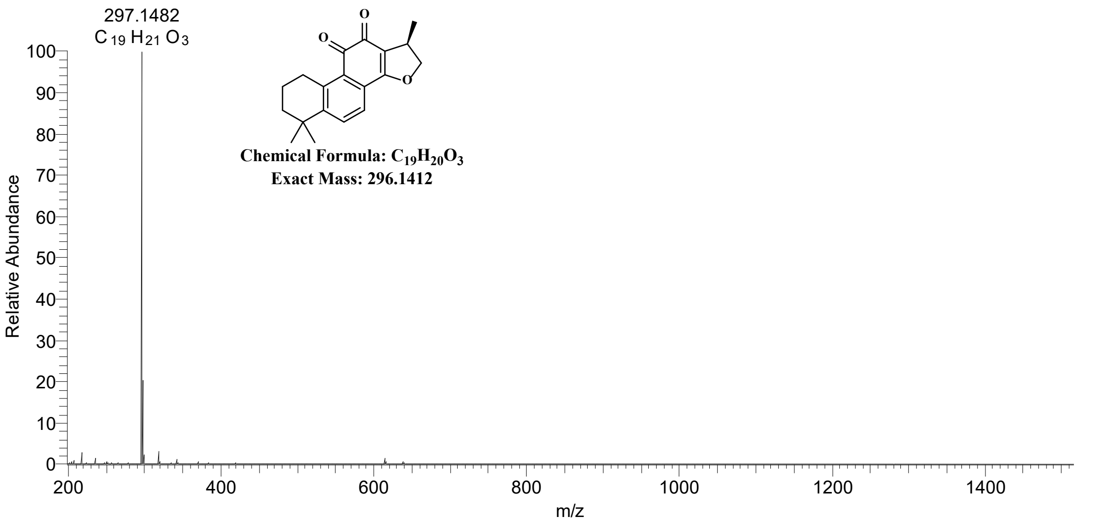

Supplementary Figure S2. Mass spectrum of F1-1. The molecular ion peak at m/z 297.1482 was consistent with the theoretical molecular weight of CTS.

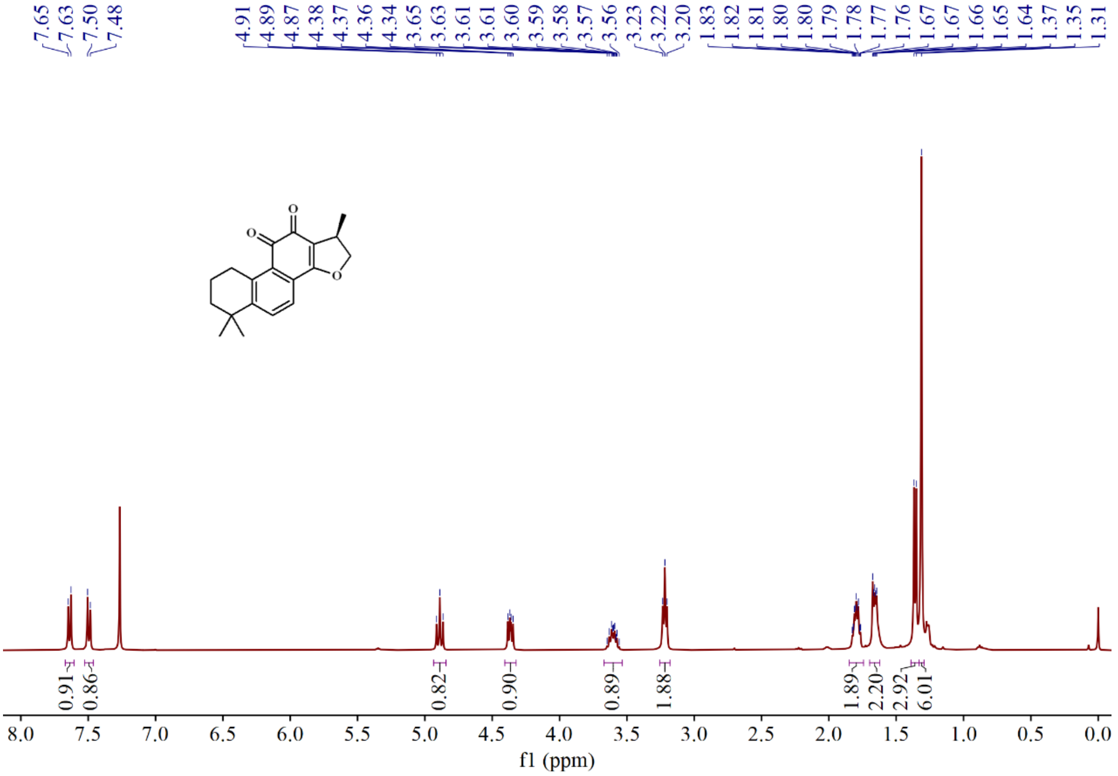

Supplementary Figure S3. ^{1}H NMR spectra of F1-1. ^{1}H NMR (400 MHz, Chloroform-*d*) δ 7.64 (d, J = 8.1 Hz, 1H), 7.49 (d, J = 8.0 Hz, 1H), 4.89 (t, J = 9.5 Hz, 1H), 4.36 (dd, J = 9.4, 6.0 Hz, 1H), 3.60 (m, 1H), 3.22 (t, J = 6.4 Hz, 2H), 1.79 (m, 2H), 1.66 (m, 2H), 1.36 (d, J = 6.8 Hz, 3H), 1.31 (s, 6H).

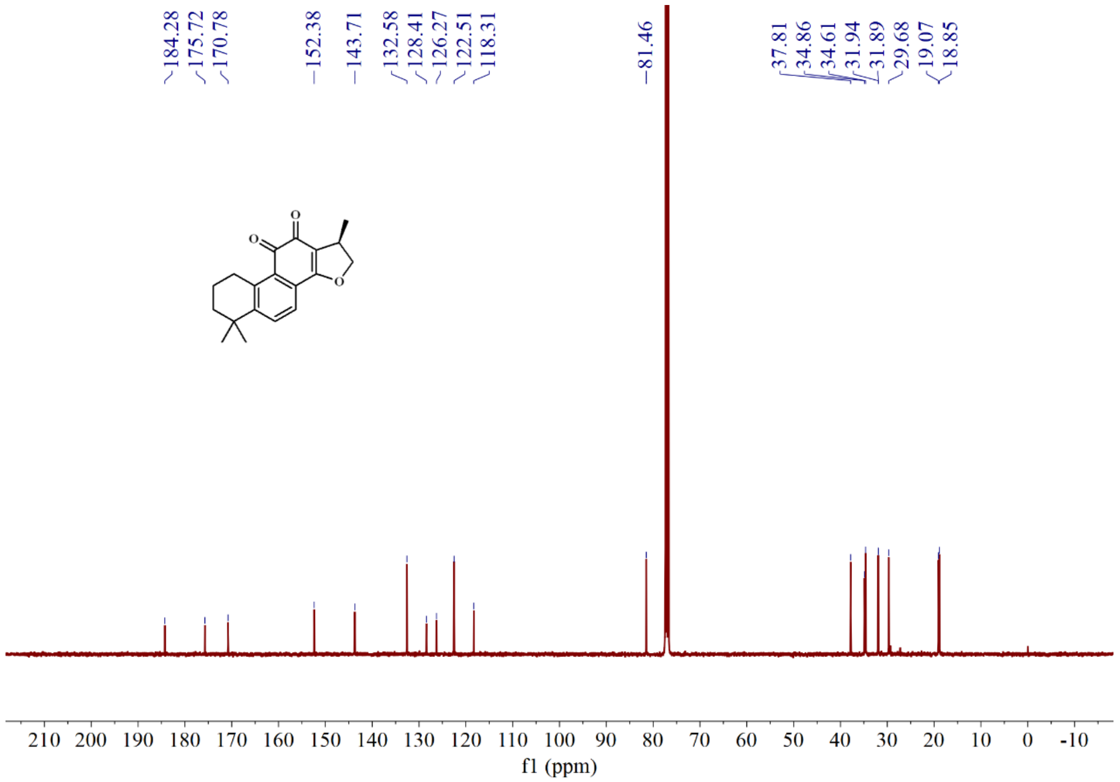

Supplementary Figure S4. ^{13}C NMR spectra of F1-1. ^{13}C NMR (101 MHz, Chloroform-*d*) δ 184.28, 175.72, 170.78, 152.38, 143.71, 132.58, 128.41, 126.27, 122.51, 118.31, 81.46, 37.81, 34.86, 34.61, 31.94, 31.89, 29.68, 19.07, 18.85.
